# Evaluation of Immunomagnetic Separation for the Detection of *Salmonella* in Surface Waters by Polymerase Chain Reaction

**DOI:** 10.3390/ijerph110909811

**Published:** 2014-09-19

**Authors:** Chao-Yu Hsu, Bing-Mu Hsu, Tien-Yu Chang, Tsui-Kang Hsu, Shu-Min Shen, Yi-Chou Chiu, Hung-Jen Wang, Wen-Tsai Ji, Cheng-Wei Fan, Jyh-Larng Chen

**Affiliations:** 1 Division of Urology, Department of Surgery, Tungs’ Taichung MetroHarbor Hospital, Taichung 435, Taiwan; E-Mail: jowyu@msn.com; 2 Department of Earth and Environmental Sciences, National Chung Cheng University, Chiayi 621, Taiwan; E-Mails: g9510820@gemail.yuntech.edu.tw (S.-M.S.); zlike123@hotmail.com (H.-J.W.); jws_bio@yahoo.com.tw (W.-T.J.); cwfan@ccu.edu.tw (C.-W.F.); 3 Department of Radiology, Cheng Hsin General Hospital, Taipei 112, Taiwan; E-Mail: jackym076@yahoo.com.tw; 4 Department of Ophthalmology, Cheng Hsin General Hospital, Taipei 112, Taiwan; E-Mail: g9510820@gemail.yuntech.edu.tw; 5 General Surgery, Surgical Department, Cheng Hsin General Hospital, Taipei 112, Taiwan; E-Mail: ejchiu3@yahoo.com.tw; 6 Department of Environmental Engineering and Health, Yuan-Pei University, Hsinchu 300, Taiwan; E-Mail: jlchen@mail.ypu.edu.tw

**Keywords:** aquatic environment, immunomagnetic separation (IMS), *Salmonella*, PCR

## Abstract

*Salmonella* spp. is associated with fecal pollution and capable of surviving for long periods in aquatic environments. Instead of the traditional, time-consuming biochemical detection, polymerase chain reaction (PCR) allows rapid identification of *Salmonella* directly concentrated from water samples. However, prevalence of *Salmonella* may be underestimated because of the vulnerability of PCR to various environmental chemicals like humic acid, compounded by the fact that various DNA polymerases have different susceptibility to humic acid. Because immunomagnetic separation (IMS) theoretically could isolate *Salmonella* from other microbes and facilitate removal of aquatic PCR inhibitors of different sizes, this study aims to compare the efficiency of conventional PCR combined with immunomagnetic separation (IMS) for *Salmonella* detection within a moderately polluted watershed. In our study, the positive rate was increased from 17.6% to 47% with nearly ten-fold improvement in the detection limit. These results suggest the sensitivity of *Salmonella* detection could be enhanced by IMS, particularly in low quality surface waters. Due to its effects on clearance of aquatic pollutants, IMS may be suitable for most DNA polymerases for *Salmonella* detection.

## 1. Introduction

Belonging to Enterobacteriaceae, *Salmonella* is a rod-shaped, Gram-negative bacterium. Many serotypes of *Salmonella* are known gastrointestinal pathogens, with some capable of causing illness in humans and other mammals with as few as 10 to 1000 organisms [1]. Contaminated foods and water are two critical infection routes for *Salmonella*. Outbreaks are frequent around the world, especially in countries with poor sanitary conditions [2]. In advanced countries like the United States, there are approximately 42,000 cases of non-typhoidal *Salmonella* infection each year [3]. Because of their strong pathogenicity and endurance in aquatic environments, epidemic strains of *Salmonella* are of considerable concern for public health [4,5]. 

Traditional methods for detecting *Salmonella* in water include selective enrichment, biochemical testing, and serological subtyping [6]. However, this process is time-consuming, laborious, and expensive [7]. Alternatively, PCR is reliable for detecting the presence and serotypes of *Salmonella* in a short time. Although highly specific, PCR is more vulnerable to environmental pollutants since concentration of bacteria by membrane filtration usually comes with residues of miscellaneous PCR inhibitors, such as humic substances, metal ions, polysaccharrides, and insoluble debris [8,9,10,11,12]. Such inhibitors may interfere with the PCR reaction via inactivating DNA polymerase or sequestering/degrading DNA templates and result in an underestimation of the *Salmonella* risk [12]. 

Several studies have suggested methods to decrease PCR inhibitors in water samples. Size exclusion chromatography was claimed helpful for removal of humic acids. The pored beads allow flow-through of large molecules like genomic DNA but detour and/or capture smaller compounds [13]. However, larger or agglutinated pollutants may be eluted simultaneously. Excessive PCR inhibitors may remain to hamper the reaction after saturation of column capacity. Besides, direct lysis of the concentrated bacteria for DNA extraction before complete removal of pollutants may expose the DNA templates to DNase secreted by environmental microbes like *Flavobacterium* or to DNA-chelating chemicals such as melanin, humic acid, and collagen [14,15]. 

Immunomagnetic separation (IMS) is a technology utilizing antibody-coated magnetic microbeads to capture specific bacteria from a variety of biological substrates including foods and feces [16,17,18,19,20]. IMS can separate the target microbe from impurities magnetically and allows efficient wash for removal of pollutants that could interfere with isolation or detection. Moreover, keeping the bacteria intact during washing further lowers the possibility of DNA degradation or sequestration. In this study, we aim to assess whether IMS could increase the detection rate of *Salmonella* by conventional PCR. By comparing with the results of quantitative PCR, the detection limits of both methods were also evaluated. 

## 2. Materials and Methods

### 2.1. Sample Collection and Pre-Treatment

**Figure 1:**
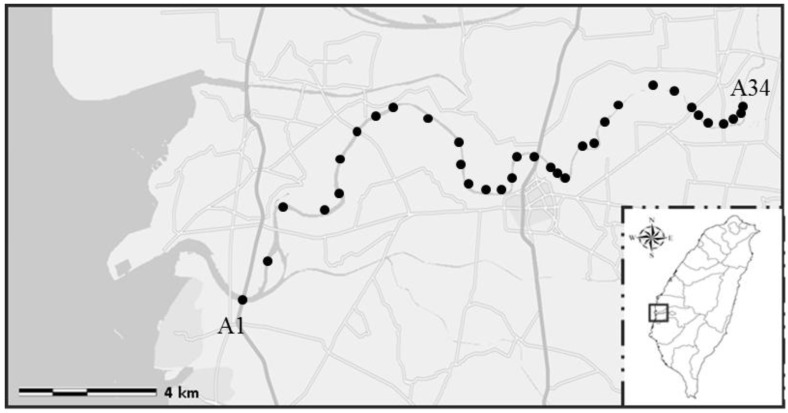
Sampling points along Puzih River. The sampling sites in the figure are shown as black dots (https://maps.google.com.tw).

**Figure 2:**
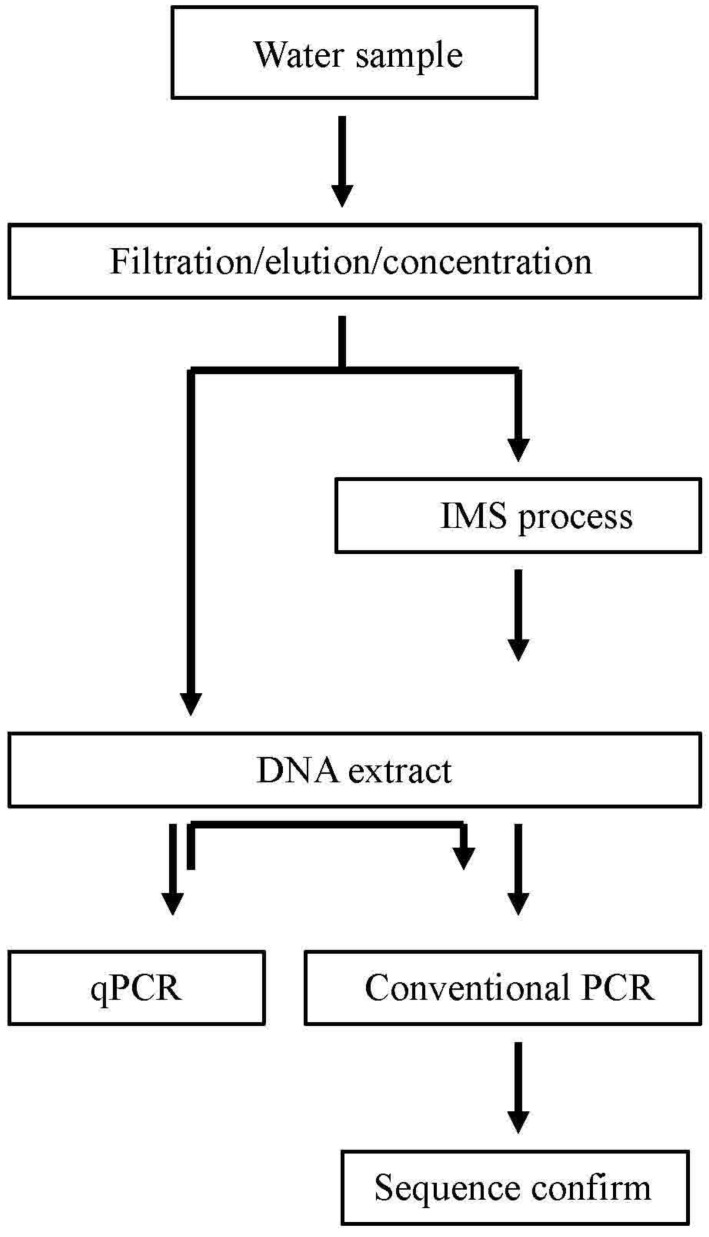
Schematic diagram of experimental setup and designated *Salmonella* detection.

The Puzih River is 75.67 km long, spanning 426.6 km^2^ in Chiayi County, mid-southern Taiwan and is an important water source for activities such as agriculture around the area. About two-thirds of the stream was declared to be polluted. Flowing through distinct geographical environments including mountainous countryside, a highly populated city, industrial zones, and costal fish farms, Puzih River has miscellaneous water pollutants ranging from household wastes (59.77%), industry (28.50%), herding or animal husbandry (7.75%), to junkyard leakage (3.98%) [21]. A total of 34 water samples were collected in March 2010 from the Puzih River (23˚28ꞌN, 120˚13ꞌE) in southern Taiwan (Figure 1). For each sample, approximately 2000 mL of water were collected in two sterile 1 L bottles, stored at 4 °C, and transported to the laboratory in 24 h. For concentration of microbes, a one liter sample water was filtered by vacuum through 45-mm diameter GN-6 membranes with a pore size of 0.22 µm (Pall, Mexico City, Mexico) in a stainless steel filter holder. The microbes captured on the surface of the membrane were then eluted by shaking and twisting membranes in 100 mL of sterile phosphate-buffered saline (137 mM NaCl, 2.7 mM KCl, 10 mM Na_2_HPO_4_, 1.8 mM KH_2_PO_4_, pH = 7.5) for 5 min for each sample. The above suspension was transferred into two conical centrifuge tubes (50 mL each) and centrifuged at 5800 g for 30 min at room temperature. After centrifugation, the top supernatant fluid (about 47.5 mL) was aspirated and discarded. The pellet in the remaining 2.5 mL solution was resuspended by vortexing. For each water sample, two tubes of 2.5 mL concentrate were produced for further analyses. For the sake of comparison, a portion of the eluate was collected for IMS processing, and the other was used for *Salmonella* detection without IMS (Figure 2). 

### 2.2. Immunomagnetic Separation (IMS) Procedure

For capturing *Salmonella*, 1 mL of concentrate was added to a 1.5-mL microfuge tube containing a 20 μL aliquot of Dynabeads coated with anti-*Salmonella* antibodies (Dynal AS, Oslo, Norway). The microfuge tube was affixed to a rotating mixer and rotated at approximately 18 rpm for 1 h at room temperature. After rotating, the microfuge tube was placed in a magnetic particle concentrator (MPC-S) for 3 min, and then the supernatant was decanted without removing the tube from the MPC-S. The bead-*Salmonella* complex was then mixed with 1 mL PBS-Tween 20 (0.1%) to wash it, after which the microfuge tube was placed in the MPC-S for 2 min. The supernatant was discarded, and the bead-*Salmonella* complex underwent the wash procedure three times, after which the bead-*Salmonella* complex was resuspended in 100 μL of PBS-Tween 20 solution. The suspension was subsequently subjected to extraction of the total bacterial genomic DNA. 

### 2.3. PCR Analysis for Salmonella and Sequence Analysis

Both suspensions (with and without IMS) underwent extraction of total genomic DNA using a DNA extraction kit (Viogene, Taipei, Taiwan) according to the manufacturer’s instructions. The quality and quantity of extracted DNA were evaluated by NanoDrop spectrometer (Thermo Scientific, Wilmington, DE, USA). The concentration of extracted DNA was evaluated as described [22]. The solution was then analyzed for the presence of *inv*A, a gene conserved in nearly all *Salmonella* serotypes, by conventional PCR and quantitative PCR (qPCR) [23]. The reaction solution for conventional PCR was prepared using 2.5 μL of the DNA templates (5 ng/μL) in conjunction with the PCR mixture containing 5 µL 10× PCR buffer (12.5 mM MgCl_2_), 0.5 µL dNTP mix (40 mM of each dNTP), 0.2 µL Qiagen Taq polymerase mix (5 units/µL), 14.8 µL DNase-free deionized water, and 1 µL of previously reported *inv*A1/*inv*A2 primers (*inv*A1: 5'-ACAGTGCTCGTTTACGACCTGAAT-3' and *inv*A2: 5'-AGACGACTGGTACTGATCGATAAT-3'; 10 mM of each primer) [16]. The total volume was 25 µL. Furthermore, the negative DNA control (template DNA replaced with distilled water) and positive control (*Salmonella* *enterica* ATCC 13076) were also analyzed during each run. For primers invA1 and invA2 the reactions were run for 35 cycles: denaturation was at 95 °C for 0.75 min, annealing at 58 °C for 1 min and extension at 72 °C for 1 min. The products were mixed with loading buffer (10 mM EDTA, 10% glycerol, 0.015% bromophenol blue, 0.17% SDS) and analyzed using gel electrophoresis on a 2% agarose gel (Biobasic Inc., Markham, ON, Canada) performed with 5 μL of the reaction solution. A 100-bp DNA ladder was used as a DNA size marker. DNA products were visualized by ethidium bromide staining (0.5 μg/mL, 10 min) and imaged under UV light. The DNA fragments of gel in target gene site (244 bp) was cut, purified and analyzed by sequencing using a Bio-Dye Terminator Cycle Sequencing Kit (Applied Biosystems, Grand Island, NY, USA). All samples and positive and negative DNA controls were analyzed in triplicates in each conventional PCR run.

### 2.4. qPCR Analysis for Salmonella

The qPCR was performed in a 20 μL reaction mixture with 10 μL SYBR^®^ Premix Ex Taq (Takara, Otsu, Japan), 4.5 μL DNase-free deionized water, 1 µL of *inv*A1/*inv*A2 primers, and 2.5 μL templates DNA from directly concentrated water samples without IMS procedure (5 ng/μL). The reaction was begun with a 10 min denaturation step at 95 °C, followed by 35 cycles of 95 °C for 15 Sec and 58 °C for 30 Sec. Nuclease-free water (Qiagen, Hilden, Germany) was used in all experiments as a negative control. Each qPCR run was conducted using standard DNA, sample DNA, and a negative control. 

For quantification, cultures of original *Salmonella* spp. were used (*Salmonella* *enterica* ATCC 13076). The bacteria suspension was ten-fold diluted and plated on Xylose-Lysine-Desoxycholate (XLD) agar for bacteria counting. A portion of the diluted suspension was used for DNA extraction simultaneously. Bacterial DNA extraction and subsequent qPCR was performed in triplicate for each dilution. After completion of qPCR, the standard curve was constructed by plotting cycle threshold value (Ct) values against the DNA concentrations from known number of *Salmonella*. The regression coefficient was kept higher than 0.99 in each experiment. The Ct in the linear range of the assay was applied to the standard curves generated previously to determine the total number of bacteria in water samples, and was then converted to CFU (colony forming unit)/mL.

### 2.5. Physical and Microbiological Parameter Analysis

Water samples (300 mL) were collected in a sampling bag (Nasco Whirl-Pak, Fort Atkinson, WI, USA) and stored at 4 °C for the determination of the heterotrophic bacterial count and analysis of total coliforms within 24 h. Heterotrophic bacterial count were measured using the spread plate method, and total coliforms by membrane filtration and incubation on a differential medium agar, as prescribed in the Standard Method for the Examination of Water and Wastewater (Methods 9215 C and 9222 B) [6]. In addition to the bacterial count, *in-situ* measurements of various water quality parameters were taken at each sampling location, including water temperature, pH level using a portable pH meter (D-24E, Horiba Co., Fukuoka, Japan), and turbidity (HACH Co., Loveland, CO, USA). The calculations of the correlations between *Salmonella* and five water quality parameters were determined using the STATISTICA software (StatSoft, Inc., Tulsa, OK, USA). 

## 3. Results and Discussion

Along the Puzih River, the most probable sources of *Salmonella* include wastewater from riverside feedlots and households. However, several environmental compounds and pollutants may interfere with the detection of *Salmonella* in the water samples by PCR. In this study, the Puzih River was chosen for evaluating the effect of IMS on detection of *Salmonella* in polluted waters. According to EPA guidelines, the quality of surface water on the basis of the total coliform count can be classified as (A), with 95th percentile value below 50 CFU/100 mL; (B), between 51 and 5000 CFU/100 mL; (C) between 5001 and 10,000 CFU/100 mL; (D), with 95th value above 10,000 CFU/100 mL; and (E), the poorest. Based on *E. coli* levels, the water quality of the Puzih River was found to fall between classes B and C, an evidence of moderate pollution (Table 1). Because water containing pollution from many sources could contain PCR inhibitors of various kinds such as organic compounds, heavy metals, and humic acids, and various commercial DNA polymerases have different vulnerability, pretesting the reaction efficiency may be required before detection of environmental pathogens [13]. To facilitate detection of water pathogens independent of the polymerases used, we examined whether IMS could improve the detection of *Salmonella* by the cheaper, highly accessible conventional PCR. 

At first, the presence of *Salmonella* was confirmed by real-time or quantitative (qPCR) using SYBR^®^ Premix Ex Taq, a frequently used reagent for environmental microbes [24,25]. As shown in Table 1 and supplementary Figure S1, *Salmonella* was detected in almost all of the water samples by qPCR (97.1% positive). The prevalence rate and the mean concentration derived from normalization to standard curves were 100.0% and 2.2 × 10^4^ CFU/100 mL in class B water bodies while 91.3% and 1.3 × 10^5^ CFU/100 mL in class C samples. Based on these findings, 1 mL water potentially contained infective doses of *Salmonella*.

Rompré reported that total coliforms could be used as an indicator for the presence of entero-pathogens [26]. In the current study, the *Salmonella* detection rates were not significantly correlated with several water quality parameters such as temperature, pH, turbidity, total coliform, and heterotrophic plate counts (Supplementary Table S1). Because environmental factors such as temperature may be similar along the stream especially in such a small rivershed, it may be difficult to see their relationship with presence of *Salmonella*. These results suggest that EPA guidelines related to the quality of surface water do not completely reflect the infection risks of contaminated waters. Therefore, a more efficient and specific method like IMS-combined conventional PCR may be helpful for pathogens detection. 

**Table 1:** The occurrence and quantification for *Salmonella* using PCR with and without IMS process.

Sample No. *	Class of Water Quality	*Salmonella* Positive of Conventional PCR		Quantification of qPCR for *Salmonella*, CFU/100 mL
		Non IMS Procedure	IMS Procedure	
A1	C	N	P	1.0 × 10^4^
A2	C	P	P	7.3 × 10^4^
A3	C	N	N	2.7 × 10^6^
A4	C	N	P	1.2 × 10^4^
A5	C	N	P	2.8 × 10^4^
A6	C	N	P	5.3 × 10^3^
A7	C	P	P	ND
A8	C	N	P	7.1 × 10^3^
A9	C	N	N	1.7 × 10^4^
A10	C	P	P	2.1 × 10^4^
A11	C	N	P	4.7 × 10^3^
A12	C	N	N	7.6 × 10^3^
A13	C	P	P	1.1 × 10^4^
A14	C	N	N	1.7 × 10^4^
A15	C	N	N	1.9 × 10^4^
A16	C	N	P	1.2 × 10^3^
A17	C	N	N	8.6 × 10^3^
A18	C	N	N	1.1 × 10^4^
A19	C	N	P	5.2 × 10^3^
A20	C	N	N	2.0 × 10^4^
A21	C	N	N	1.8 × 10^4^
A22	C	N	P	1.9 × 10^4^
A23	C	N	P	1.2 × 10^4^
A24	B	N	N	3.1 × 10^3^
A25	B	N	P	1.0 × 10^4^
A26	B	N	P	5.5 × 10^4^
A27	B	P	N	2.0 × 10^4^
A28	B	N	N	3.4 × 10^4^
A29	B	N	N	8.1 × 10^4^
A30	B	P	N	1.1 × 10^4^
A31	B	N	N	7.9 × 10^3^
A32	B	N	N	1.9 × 10^4^
A33	B	N	N	4.6 × 10^3^
A34	B	N	N	2.4 × 10^2^

Notes: * The numbering of samples A1–A34 represents locations along Puzih River. N: *Salmonella* negative. P: *Salmonella* positive. ND: not determined. B: Total coliform count between 51 and 5000 CFU/100 mL. C: Total coliform count between 5001 and 10,000 CFU/100 mL.

Although qPCR could detect *Salmonella* in nearly all the water samples, conventional PCR using Qiagen Taq polymerase could detect *Salmonella* in a few samples. Without IMS, *Salmonella* was only detected in six of the 34 samples (17.6%) by conventional PCR, suggesting the sensitivity of qPCR is much better. With the inclusion of IMS, *Salmonella* was detected in 16 of the 34 samples (47.1%). In other words, twelve samples that were negative without IMS were positive with IMS, although two of the positive samples, A27 and A30, were negative after IMS was used. According to the results of qPCR, *Salmonella* levels in samples A27 and A30 seem not obviously lower than the other detectable ones. Why the microbe could not be detected after IMS may be due to loss of bacteria after harsh washing conditions although the small sample size of class B may be not representative [27]. However, in class C samples the detection rate increased from 17.4% to 60.9% after IMS. The detection limit for *Salmonella* detection by conventional PCR improved from 1.1 × 10^4^ CFU/100 mL to 1.2 × 10^3^ CFU/100 mL when compared with qPCR, although in some samples higher concentrations of *Salmonella* were not detected possibly because of humic acids. To confirm IMS procedure has better detection rate, we tested whether the result of IMS has relationship with the one of IMS-independent procedure (Supplementary Table S2). If these two procedures have similar positive effects, theoretically they will have linkage after Chi test. The result suggests no relationship (*p* = 0.289) and confirms the independence between these two procedures. The independence supports the conclusion that IMS has better detection rate. These results suggest the application of IMS can improve the efficiency of conventional PCR for detection of *Salmonella*. Interestingly, the A7 sample was determined positive by conventional PCR independently of IMS while it was not detected by qPCR. Because SYBR Green was confirmed to hamper PCR, we speculate that in aquatic samples some unknown chemicals could exacerbate the problem and thus increase the difficulty in quantification by qPCR [28]. Hence, conventional PCR in combination with IMS is still helpful in detection of aquatic pathogens. 

With the fast growing number and variety of environmental pollutants, it is difficult to predict the efficacy of reported methods or reagents in all the cases of polluted waters. IMS may facilitate removal of most residues after direct concentration of environmental microbes. Although IMS could enhance the detection of *Salmonella* in our study, De Medici *et al.* claimed little effect of IMS on the detection of pre-enriched *Salmonella* in poultry by SYBR Green I qPCR [29]. It seems that IMS is not essential for pathogen detection in samples with little contamination of humic acid. In our results IMS similarly had no additive effects during detection of aquatic *Salmonella* by SYBR^®^ Premix Ex Taq, suggesting IMS may be dispensable for humic acid-tolerable polymerases (data not shown). It is also possible that dense impurities may interfere with the binding between *Salmonella* and the immunomagnetic beads. Free proteases could also degrade the coated antibodies. Some modifications in sample pretreatment procedures, for example the addition of protease inhibitors, may further improve the applicability of IMS in highly polluted waters. However, many studies have shown the value of IMS due to its specificity and sensitivity during detection of pathogens in meats and other foods [30,31,32,33]. Although PCR is a good alternative to traditional culture and serotyping, it is more vulnerable to environmental pollutants. According to our data, IMS significantly improves the efficiency of conventional PCR on detection of directly concentrated *Salmonella*. The results suggest the suitability of IMS for sample pretreatment for PCR using several DNA polymerases. In conclusion, IMS is a considerable choice for detection of *Salmonella* in surface waters. 

## 4. Conclusions 

The PCR assay can be used to detect *Salmonella* in aquatic environments, however, accompanying impurities often interfere with the reaction. In this study, we showed that IMS significantly increased the efficiency of conventional PCR on detection of directly concentrated *Salmonella*. Our results suggested IMS is applicable for identifying pathogens in samples likely contaminated with various water pollutants. Taken together, IMS combined with PCR is a highly specific and sensitive method for the rapid detection of *Salmonella* in environmental water samples. 

## Supplementary Files

Supplementary File 1
