# Supplementary material for: Evaluation of Immunomagnetic Separation for the Detection of Salmonella in Surface Waters by Polymerase Chain Reaction
Source: Int J Environ Res Public Health. 2014 Sep 19;11(9):9811–21. doi: 10.3390/ijerph110909811 (PMC4199051; doi:10.3390/ijerph110909811)
Supplement: Supplementary File 1 [file ijerph-11-09811-s001.pdf]

Supplementary Information

Evaluation of Immunomagnetic Separation for the Detection of Salmonella in Surface Waters by Polymerase Chain Reaction

Table S1. Nonparametric test results for Salmonella in terms of water quality parameters.

Water Quality Parameters	Salmonella Positive	Negative	Mann-Whitney (U Test)
Heterotrophic plate counts (CFU/mL)	$2.0 \times 10^4 \pm 2.4 \times 10^{4\#}$	$5.0 \times 10^4 \pm 3.5 \times 10^2$	0.079
Total coliforms (CFU/100 mL)	$2.3 \times 10^4 \pm 2.7 \times 10^4$	$5.4 \times 10^4 \pm 2.5 \times 10^4$	0.107
Turbidity (NTU)	96.3 ± 30.4	69.4 ± 82.9	0.826
Temperature (℃)	21.3 ± 0.8	21.3 ± 0.3	0.942
pH value	7.9 ± 0.2	7.9 ± 0.3	0.883

Note: # Mean ± SD.

Table S2. The detection results of two procedures.

Detection Result		With IMS Positive	With IMS Negative
Without IMS	Positive	4	2
	Negative	12	16

Figure S1. Positive samples of the procedures with and without IMS. The bacteria quantity in each sample was also shown.

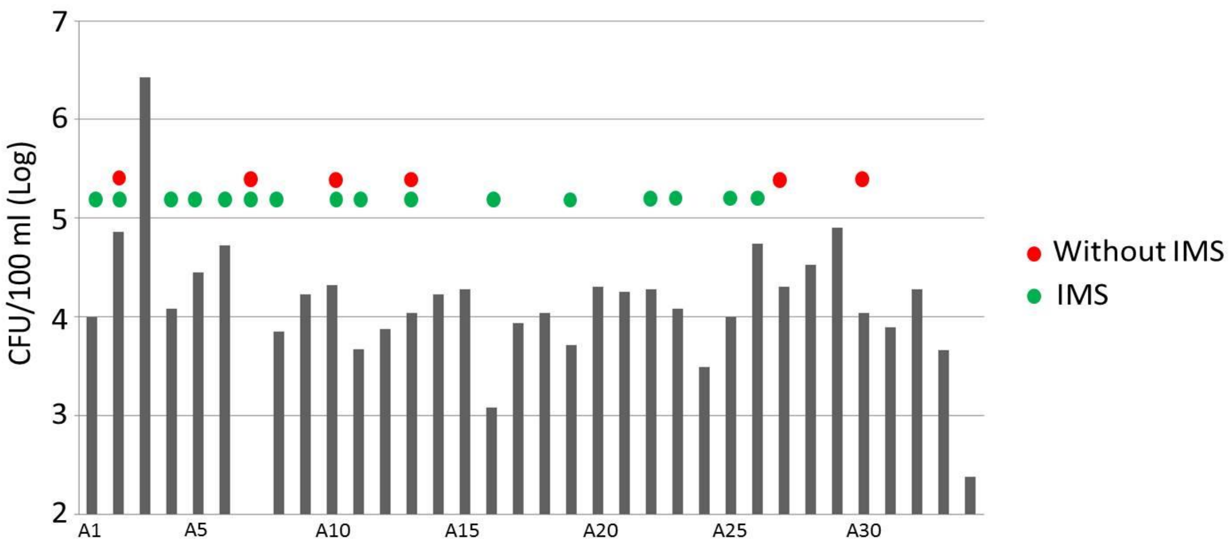

© 2014 by the authors; licensee MDPI, Basel, Switzerland. This article is an open access article distributed under the terms and conditions of the Creative Commons Attribution license (http://creativecommons.org/licenses/by/3.0/).
